# White donor, younger donor and double lung transplant are associated with better survival in sarcoidosis patients

**DOI:** 10.1038/s41598-018-25144-x

**Published:** 2018-05-03

**Authors:** Oriana Salamo, Shiva Roghaee, Michael D. Schweitzer, Alejandro Mantero, Shirin Shafazand, Michael Campos, Mehdi Mirsaeidi

**Affiliations:** 1 Section of Pulmonary, Miami VA Healthcare System, Miami, FL USA; 2 Department of Public Health, Division of Biostatistics, University of Miami, Miami, FL USA; 3 Division of Pulmonary and Critical Care, University of Miami, Miami, FL USA

## Abstract

Sarcoidosis commonly affects the lung. Lung transplantation (LT) is required when there is a severe and refractory involvement. We compared post-transplant survival rates of sarcoidosis patients with chronic obstructive pulmonary disease (COPD) and idiopathic pulmonary fibrosis (IPF). We also explored whether the race and age of the donor, and double lung transplant have any effect on the survival in the post transplant setting. We analyzed 9,727 adult patients with sarcoidosis, COPD, and IPF who underwent LT worldwide between 2005–2015 based on United Network for Organ Sharing (UNOS) database. Survival rates were compared with Kaplan-Meier, and risk factors were investigated by Cox-regression analysis. 469 (5%) were transplanted because of sarcoidosis, 3,688 (38%) for COPD and 5,570 (57%) for IPF. Unadjusted survival analysis showed a better post-transplant survival rate for patients with sarcoidosis (*p* < 0.001, Log-rank test). In Cox-regression analysis, double lung transplant and white race of the lung donor showed to have a significant survival advantage. Since double lung transplant, those who are younger and have lower Lung Allocation Score (LAS) at the time of transplant have a survival advantage, we suggest double lung transplant as the procedure of choice, especially in younger sarcoidosis subjects and with lower LAS scores.

## Introduction

Sarcoidosis is a multiorgan granulomatous disorder of unknown etiology associated with significant morbidity and mortality. The lung is the organ most commonly involved and the disease is characterized by frequent pulmonary exacerbations and remissions, particularly within the first 2 to 5 years of the diagnosis^1,2^.

Sarcoidosis can affect all age groups but is more frequent in middle age populations, and women are two times more likely to have the disease^3^. There is a known difference in prevalence according to race, with Caucasians having a 0.85% lifetime risk of developing sarcoidosis, while this risk for African Americans is 2.5%^4^. Racial differences in disease severity are also observed. African Americans have a more acute and severe presentation in contrast to a typically slow insidious process in European Americans^5^.

Medical treatment of symptomatic sarcoidosis is based on immunomodulation. Corticosteroids are usually the first line of therapy, but other medications including methotrexate, leflunomide, infliximab and other anti-TNF agents are commonly used^6^. The selection of the best treatment option is based on the degree of lung involvement, ranging from mild interstitial pulmonary disease to severe disabling pulmonary fibrosis and pulmonary hypertension^3^. Eligible patients with severe lung involvement, refractory to medical therapy, are referred for lung transplantation (LT).

Since the first successful lung transplant was performed in 1983, thousands procedure have been performed worldwide^7^. Lung transplantation has been recommended in patients with advanced chronic obstructive pulmonary disease (COPD), bronchiectasis, cystic fibrosis (CF), primary pulmonary hypertension, idiopathic pulmonary fibrosis (IPF), sarcoidosis, and other end stage interstitial lung diseases^8^. Advanced COPD and IPF are the most common indications for lung transplants worldwide^7^. Advances in lung transplant techniques, post-transplant patient care, and immunosuppressive therapies during the last 30 years have significantly improved survival^9^. The median survival of adults who undergo lung transplantation is reported to be 5.6 years, with an unadjusted survival rate of 53% at 5 years, and 31% at 10 years^10^. The highest post-lung transplant survival rate per specific disease occurs in CF patients, with a 67% survival rate at 5 years^11^. Meanwhile 5 year survival is reported to be 45% for COPD and 44% for IPF patients^12^. There are limited information regarding survival rates following LT in patients with sarcoidosis^13^. Taimeh *et al*. recently found no difference in lung transplant outcomes between sarcoidosis and non-sarcoidosis lung transplant recipients in the last 30 years^14^. However, given the significant improvement in LT outcome in the last 10 years, the sarcoidosis survival after LT may have also been improved. Moreover, information on the factors that influence on sarcoidosis survival in lung transplantation are still unclear. The aim of this study was to compare the post-transplant survival rates of sarcoidosis patients with the two most common indications for LT: COPD and IPF. We also explore whether some independent factors, such as race of the donor, age of the donor and double lung transplant have any effect on the survival of sarcoidosis patients in the post transplant setting.

## Materials and Methods

We analyzed adult sarcoidosis subjects who underwent LT in the United States from December 30^th^, 2005, to December 30^th^, 2015 based on the United Network for Organ Sharing (UNOS)/OPTN standard transplant analysis and research files database. All methods were conducted in accordance with pertinent guidelines and regulations. We used open resource de-identified national dataset, therefore no Institutional Review Board (IRB) or informed consent was required. From the same dataset, all subjects with diagnosis of COPD and IPF were selected for survival rate comparisons. The variables of interest were age, sex, race, body mass index (BMI), and outcomes including death and re-transplantation.

A log-rank test was utilized to determine if there were any statistically significant differences in survival between the three groups of interest (*p* < 0.05) and Kaplan-Meier curves were plotted to depict ordinality of any found differences. A cox regression model was performed to find if variables including age of transplant recipient, age of donor, race of donor, double lung transplant, Lung Allocation Score (LAS), cold ischemic time (time of removal of the organ from the donor to the time the organ is transplanted into the recipient), mechanical ventilation, and male gender are independently associated with mortality in sarcoidosis subjects. We developed multivariate analysis model based on variables which have previously been used by Taimeh’s *et al*.^14^. Factors were selected based on clinically important variables which was found in previous study. Missing data in the 10-year dataset was also studied, and collinearity between covariates was also explored. A second cox regression model was performed only in the overall black population to determine if the race of the donor had a persistent effect on the outcome. Moreover, a separate cox regression model was performed in non-white patients. Adjusted hazard ratio (HR) and 95% confidence interval (CI) were estimated. All analyses were performed in R (version 3.2.2) and the Survival package (version 2.38-3) and results were considered significant at *P* < 0.05.

## Results

A total of 9727 patients with sarcoidosis, COPD and IPF underwent LT from 2005 to 2015. Of these, 469 (4.8%) were transplanted because of advanced sarcoidosis, 3688 (37.9%) for COPD and 5570 (57.3%) for IPF.

Table 1 summarizes the demographic characteristics of transplanted subjects with sarcoidosis, COPD, and IPF. Age at time of transplantation for sarcoidosis was 52.91 ± 8.29 years, showing statistical difference with that of COPD (60.92 ± 6.20 years; *p* =  < 0.0001) or IPF (60.91 ± 8.60 years; *p* =  < 0.0001). About half of the sarcoidosis subjects were female, similar to what was observed in COPD but not in IPF, where an overall significant difference was observed (*p* =  < 0.0001), as most IPF subjects were male. In the sarcoidosis group, 64% (302) of the subjects were African American while 90% (3346) and 82% (4582) were European American in the COPD and IPF group, respectively. Finally, lung re-transplant rates were relatively low but statistically significantly higher in patients with IPF.

**Table 1:** Demographic features of patients who undergone lung transplant for COPD, IPF and sarcoidosis in the US in from December 2005 through December 2015.

	Total n = 9727	Sarcoidosis n = 469 (4.8%)	COPD n = 3688 (37.9%)	IPF n = 5570 (57.3%)	*p* value	
					*	**
**Age, Year, mean ± SD**	60.53 ± 7.95	52.91 ± 8.29	60.92 ± 6.20	60.91 ± 8.60	< 2.2e-16	< 2.2e-16
**Female n (%)**	3469(35.7%)	218(46.5%)	1766(47.9%)	1485(26.7%)	<2.2e-16	
**Race**					0.0004998	
Hispanic/Latino	533	8	49	476		
White	8075	147	3346	4582		
Black	912	302	263	347		
Asian	143	7	11	125		
American-Indian/Alaska Native	25	0	5	20		
Native-Hawaiian/other	6	0	0	6		
pacific islander	33					
Multiracial	0	9	14	14		
Unknown		0	0	0		
**BMI, mean ± SD**	26.01 ± 4.22	25.58 ± 4.30	24.45 ± 4.10	27.08 ± 3.96	1.014e-07	1.158e-12
**Re-transplant**	204(2.1%)	9(1.9%)	54(1.5%)	141(2.5%)	0.002046	

*Sarcoidosis compared to COPD.

**Sarcoidosis compared to IPF.

**Figure 1:**
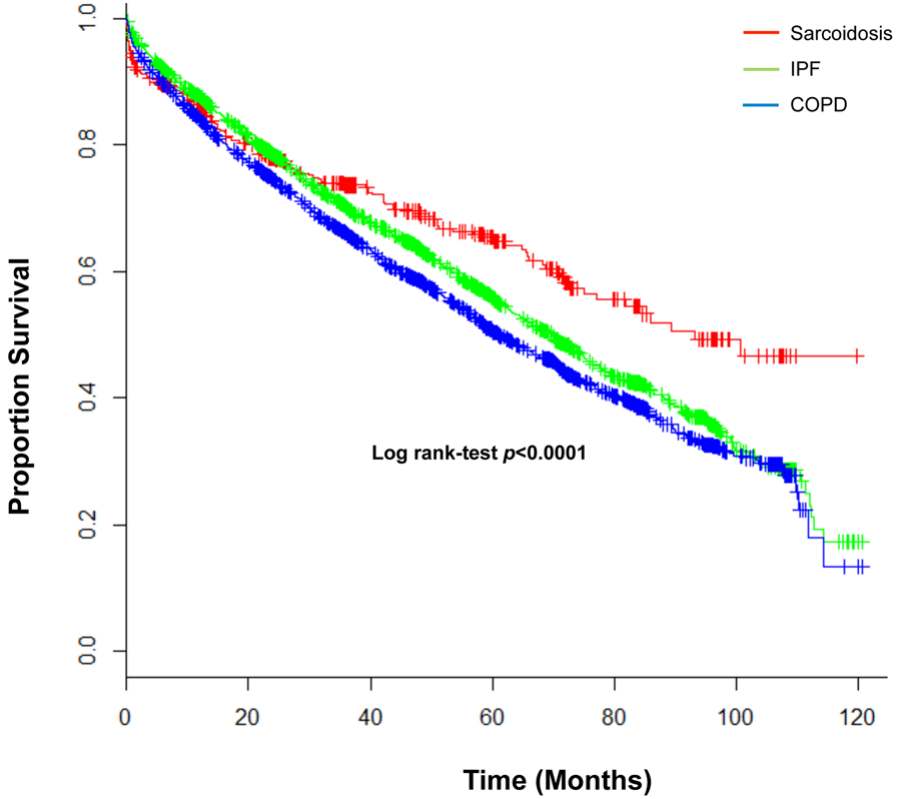
Kaplan Meier curves displaying survival rates between Sarcoidosis, COPD and IPF groups.

**Figure 2:**
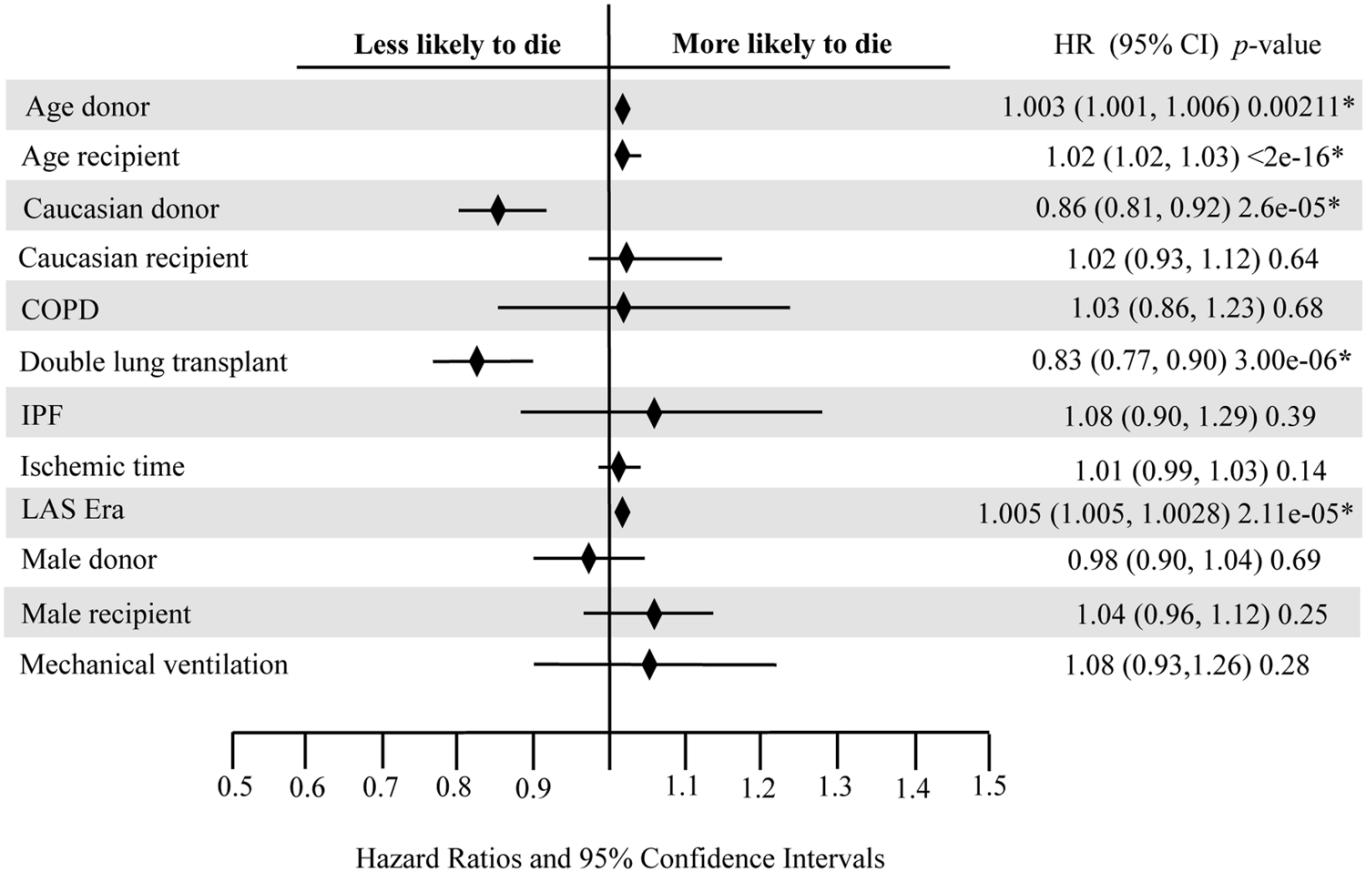
Forest plot representing independent factors associated with mortality in patients with sarcoidosis who undergone lung transplantation.

**Figure 3:**
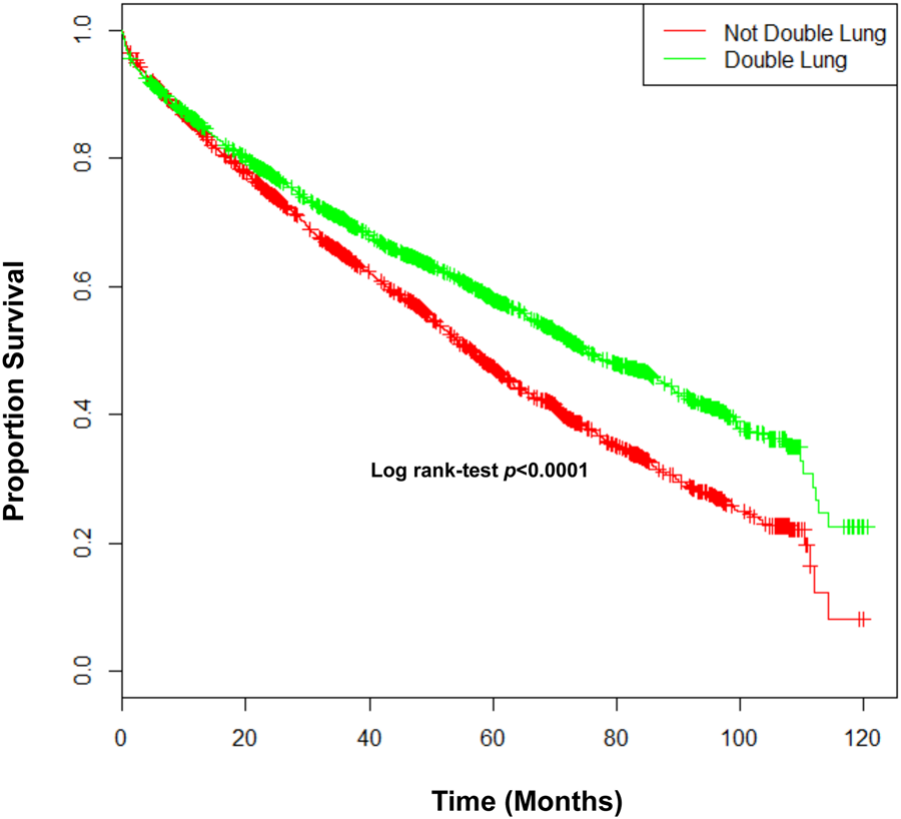
Kaplan Meier curves displaying post-transplant survival rates among not double lung and double lung transplant.

The survival analysis in the three groups showed a better survival rate for patients with sarcoidosis compared with patients with COPD and IPF (*p* < 0.001, log-rank test). Figure 1 displays the significant post-transplant survival difference between sarcoidosis and the COPD and IPF groups. In Cox-regression analysis, double lung transplant and white race of the lung donor showed to have a significant survival advantage, with an HR of 0.83 (95%CI: 0.77–0.90) and 0.86 (95%CI: 0.81–0.92), respectively. Figure 2 illustrates a plot of the estimates and the 95% confidence intervals obtained from Cox regression analysis representing independent factors associated with mortality in patients with sarcoidosis who underwent lung transplantation. Moreover, higher age of both lung- recipient and -donor plus higher LAS were independently associated with increased risk of mortality among the sarcoidosis group. Figure 3 represents Kaplan-Meier curves where a significant post-transplant survival difference is appreciated in the double lung transplant group (*p* < 0.001, log-rank test).

A second Cox-regression model was made only in African American subjects to explore if the Caucasian donor effect persisted. In this model we couldn’t find any significant differences concerning the impact of the race of the donor on the survival rate. Moreover, as illustrated in Table 2, male donor and higher age of the donor were associated with a negative outcome in this population.

**Table 2:** Cox regression model in African Americans who undergone lung transplant for COPD, IPF and sarcoidosis in the US in from December 2005 through December 2015.

Variable	Hazard Ratio	Lower 95%	Upper 95%	*p* value
Double lung	1.0625	0.8097	1.394	0.6617
COPD	1.0880	0.8027	1.475	0.5867
IPF	1.2449	0.9506	1.630	0.1115
Age (recipient)	1.0066	0.9936	1.020	0.3190
Male (recipient)	0.9821	0.7685	1.255	0.8855
Mechanical ventilation	2.0176	0.9971	4.082	0.0510
Ischemic time	1.0167	0.9471	1.091	0.6467
Caucasian (donor)	0.8642	0.6925	1.078	0.1966
Male (donor)	1.3343	1.0302	1.728	**0.0289**
Age (donor)	1.0079	1.0000	1.016	**0.0490**
LAS Era	1.0023	0.9946	1.010	0.5576

A third Cox-regression model was made in the non-white population. We couldn’t find any impact concerning the race of the donor regarding survival. Significant statistical differences were found in recipients and donors with higher age, being associated with poor outcome, as showed in Table 3. It is important to mention that LAS score was not longer significantly associated anymore. This may suggest that the scoring system needs further investigation for its effectiveness to rank non-white patients, including African Americans.

**Table 3:** Cox regression model in non-white subjects who undergone lung transplant for COPD, IPF and sarcoidosis in the US in from December 2005 through December 2015.

Variable	Hazard Ratio	Lower 95%	Upper 95%	*p* value
Double lung	1.0228	0.8440	1.239	0.81821
COPD	1.0415	0.7938	1.366	0.76935
IPF	1.1241	0.8944	1.413	0.31574
Age (recipient)	1.0125	1.0028	1.022	**0.01100**
Male (recipient)	1.0497	0.8760	1.258	0.59933
Mechanical ventilation	1.1637	0.7514	1.802	0.49694
Ischemic time	1.0457	0.9917	1.103	0.09855
Caucasian (donor)	0.9556	0.8100	1.127	0.58989
Male(donor)	1.0585	0.8820	1.270	0.54148
Age (donor)	1.0095	1.0035	1.015	**0.00188**
LAS Era	1.0006	0.9950	1.006	0.83991

### Collinearity

**Figure 4:**
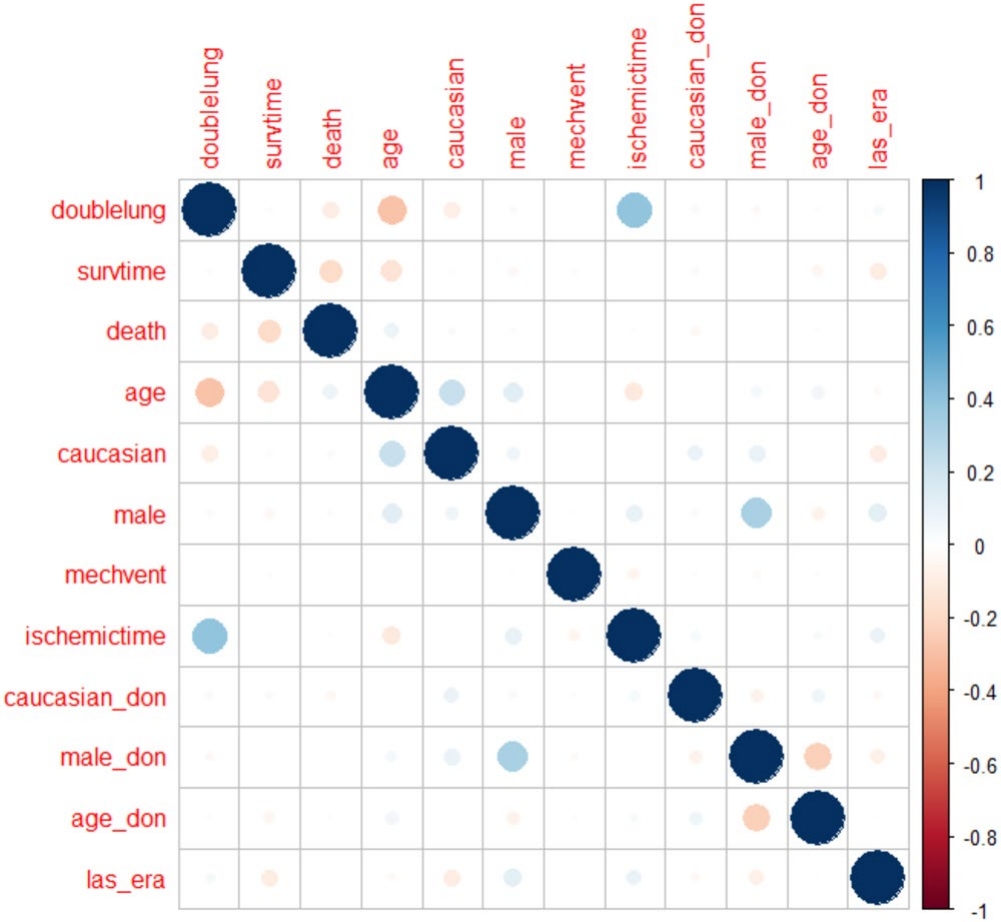
Collinearity between covariates.

Despite age being a component of LAS score, no strong collinearities were detected between these variables that would impede the estimation of this model, as shown in Fig. 4.

### Handling Missing data

Table 4 displays the missing data in the 10-year dataset. Of note, the highest rate of missing for any single variable was about 3% out of approximately 10,000 observations in the 10-year dataset. This suggests that a very complete data was utilized in the study.

**Table 4:** Missing data in dataset from who undergone lung transplant for COPD, IPF and sarcoidosis in the US in from December 2005 through December 2015.

Variable	Missing (%)
Double lung	0 (0%)
Disease	0 (0%)
Age (recipient)	0 (0%)
Caucasian (recipient)	0 (0%)
Male (recipient)	0 (0%)
Mechanical ventilation	167 (1.60%)
Ischemic time	316 (3.03%)
Caucasian (donor)	0 (0%)
Male (donor)	0 (0%)
Age (donor)	0 (0%)
LAS Era	291 (2.79%)
Survival time	21 (0.20%)

## Discussion

This 10-year lung-transplant database review of patients with a primary diagnosis of pulmonary sarcoidosis in the US shows there is a higher number of females and African American patients who underwent lung transplant due to sarcoidosis compared to IPF. There are also a higher number of African Americans in the sarcoidosis group in contrast to the COPD group. This study demonstrates that sarcoidosis subjects had a significantly higher survival rate compare to patients with COPD and IPF in an adjusted survival analysis.

Sarcoidosis comprised only a 4.8% of subjects when compared to other transplant indications, such as COPD and IPF in the last 10 years. It has not been changed in the last 30 years^14^. The high prevalence of COPD and higher severity of pulmonary fibrosis in IPF may explain such a high proportion of LT in the US. The prevalence of sarcoidosis in the United States is estimated at 1 to 40 per 100,000 people^15^, while COPD is reported to occur in as high as 15.2% of the US population^16^. The IPF prevalence is less than sarcoidosis (8.2 cases per 100, 000 people in 2010) in the US^17^, However, IPF causes severe pulmonary fibrosis and advanced lung disease in almost all subjects, while fibrotic lung disease develops in up to 20% of patients with sarcoidosis^18^.

The significant higher prevalence of females and African Americans in the sarcoidosis group highlights the gender and race disparities. Females and African Americans are more susceptible to sarcoidosis for still unknown reasons. African Americans also suffer from higher severity of disease and have a higher mortality rate^19^. Recently, we reported that the overall mortality rate of sarcoidosis was eight times higher in African Americans than in European Americans^5^.

In contrast with our study, Taimeh *et al*. could not demonstrate any association between donor age and race, and survival in the sarcoidosis cohort^14^. We can hardly compare these studies together for several reasons. We analyzed the outcomes of lung transplant in the last 10 years to achieve the current status. We also aimed to compare sarcoidosis patients who underwent lung transplantation with the two most common causes for lung transplant: COPD and IPF. Taimeh *et al*. analyzed a 30-year cohort for their analysis, comparing sarcoidosis to all non-sarcoidosis subjects. In the last 30 years, significant improvement in outcome of lung transplant has been achieved including surgical techniques, post-operative care and immunosuppressive therapy. Those factors may affect the outcome results between two studies. We considered this may be a major driven factor to see an association between donor age and race with a better outcome in sarcoidosis subjects in the current study.

Our observation of 53% survival of sarcoidosis subjects at 10 years post-lung transplant is better than previously report by Walker *et al*.^20^ that was 56% at 5 years. Interestingly, sarcoidosis subjects had a better unadjusted survival compared to COPD and IPF subjects in Kaplan–Meier analysis. The Cox regression analysis revealed that better survival in sarcoidosis subjects was secondary to their younger age, higher number of double lung transplant and receiving lungs from European-American donors. Sarcoidosis patients were on average 8 years younger than their COPD and IPF counterparts, and this was an independent risk factor in the adjusted survival analysis. The effect of age on lung transplant outcome has been studied before. Gutierrez and colleagues showed that subjects with advanced age who underwent lung transplant have an increased mortality rate even after adjusting for expected advanced age-related mortality^21^. Our study also showed that age and race of donors are independently associated with post transplant survival in sarcoidosis subjects. Sommer and colleagues could not find significant difference in 1-year survival rate when using of donor lungs of aged population (≥70 years old). But, noted significantly lower forced expiratory in 1 second values in subjects with lung transplantation of aged donor lungs in IPF recipients^22^. Obviously, our 10 years follow up study has higher power to show survival effect of aged donor lungs on post transplantation survival.

Recipients of lungs from Caucasian donors had better survival. We speculate that socioeconomic factors influence on this observation. To our knowledge, no other studies have reported a survival advantage related to race and ethnicity of donor lungs. However, studies in kidney transplant patients have found that recipients of African American kidney donors had lower graft survival^23^. Further investigation is needed to determine the nature of the relationship between donor and recipient race and survival in LT.

Our analysis showed that higher lung allocation scores are associated with worse post-LT outcomes in sarcoidosis. LAS has been universally used to prioritize LT candidates by balancing expected post-transplant survival and predicted waitlist mortality^24^. According to our analyses, sarcoidosis patients with a lower LAS at the time of transplant should have a higher post-LT survival. Therefore, a new scoring system should be developed to determine the most appropriate time for lung transplantation for patients with advanced refractory sarcoidosis.

Double lung transplant was associated with higher survival in our study. It was performed in higher percentage in sarcoidosis subjects compared to IPF and COPD, perhaps due to severe bilateral lung involvement, presence of bronchiectasis, and pulmonary hypertension. Our study suggests that double lung transplantation should be the procedure of choice in sarcoidosis patients.

Sarcoidosis recurrence after lung transplantation is an important topic. We reported no significant increasing risk for re-transplantation among sarcoidosis subjects when compared to others. The rate of re-onset of sarcoidosis after lung transplant was reported by Banga A *et al*. previously^25^. They found granulomatous reaction recurrence in 7 (23%) out of 30 sarcoidosis subjects who undergone lung transplant in Cleveland Clinic, Ohio, USA. However, they found no impact of granulomas recurrence on overall outcomes. Schultz and colleagues also reported that 7 (28%) of 25 subjects who undergone lung transplant for sarcoidosis in Copenhagen, Denmark showed recurrence of granulomas in new lungs with no impact of outcomes^26^. UNOS does not provide information concerning recurrence of sarcoidosis in post-transplanted patients. Therefore, we would not able to analyze rate of sarcoidosis recurrence after lung transplantation. This topic requires further investigation.

This study consists of a big cohort comprised of 9,727 patients that underwent lung transplant because of sarcoidosis, COPD and IPF, in a period of 10 years. To our knowledge, this is the first time that the role of the ethnicity of the donor was analyzed as an independent factor for the outcome in lung transplantation.

### Limitations

Despite the use of the largest available database for lung transplantation, our study has several limitations. We are unable to analyze the impact of many other variables that play a role in post lung transplant survival including nutritional status, psychological factors, adherence to medications, pre existent pulmonary arterial hypertension (PAH), spirometric values including total lung capacity (TLC), and other co-morbidities. Even though PAH may be a potential cofounder in post lung transplant outcome, we had no access to PAH data of subjects from UNOS database. Moreover, only a total of 912 subjects were African-American among 9,727 patients (9.4%). This represents an important limitation because we were not able to analyze the correlation between African-American recipients undergoing lung transplant from African-American donors. A more powered sample size is required to further investigate the impact of other ethnicities in the outcome of lung transplant.

Sarcoidosis patients have a reasonable survival rate and re-transplant rate that are comparable with other common LT indications such as COPD and IPF. Given that double lung transplant is associated with better survival in sarcoidosis subjects, and those who are younger and have a lower LAS score at the time of transplant have a survival advantage, we would suggest double lung transplant as the procedure of choice, especially in younger sarcoidosis subjects and preferably those with lower LAS scores.

## References

[1] Mirsaeidi M (2016). Plasma metabolomic profile in fibrosing pulmonary sarcoidosis. Sarcoidosis Vasc Diffuse Lung Dis.

[2] Mortaz E (2015). Association of serum TNF-alpha, IL-8 and free light chain with HLA-DR B alleles expression in pulmonary and extra-pulmonary sarcoidosis. J Inflamm (Lond).

[3] Newman LS, Rose CS, Maier LA (1997). Sarcoidosis. N Engl J Med.

[4] Baughman RP (2016). Sarcoidosis in America. Analysis Based on Health Care Use. Ann Am Thorac Soc.

[5] Mirsaeidi M, Machado RF, Schraufnagel D, Sweiss NJ, Baughman RP (2015). Racial difference in sarcoidosis mortality in the United States. Chest.

[6] Korsten P, Mirsaeidi M, Sweiss NJ (2013). Nonsteroidal therapy of sarcoidosis. Current opinion in pulmonary medicine.

[7] Yusen RD (2015). The Registry of the International Society for Heart and Lung Transplantation: Thirty-second Official Adult Lung and Heart-Lung Transplantation Report–2015; Focus Theme: Early Graft Failure. J Heart Lung Transplant.

[8] Orens JB (2006). International guidelines for the selection of lung transplant candidates: 2006 update–a consensus report from the Pulmonary Scientific Council of the International Society for Heart and Lung Transplantation. J Heart Lung Transplant.

[9] Watson CJ, Dark JH (2012). Organ transplantation: historical perspective and current practice. Br J Anaesth.

[10] Puri V, Patterson GA, Meyers BF (2015). Single versus bilateral lung transplantation: do guidelines exist?. Thorac Surg Clin.

[11] Stephenson AL (2015). Clinical and demographic factors associated with post-lung transplantation survival in individuals with cystic fibrosis. J Heart Lung Transplant.

[12] Titman A, Rogers CA, Bonser RS, Banner NR, Sharples LD (2009). Disease-specific survival benefit of lung transplantation in adults: a national cohort study. Am J Transplant.

[13] Nathan SD (2005). Lung transplantation: disease-specific considerations for referral. Chest.

[14] Taimeh Z, Hertz MI, Shumway S, Pritzker M (2016). Lung transplantation for pulmonary sarcoidosis. Twenty-five years of experience in the USA. Thorax.

[15] Erdal BS, Clymer BD, Yildiz VO, Julian MW, Crouser ED (2012). Unexpectedly high prevalence of sarcoidosis in a representative U.S. Metropolitan population. Respir Med.

[16] Adeloye D (2015). Global and regional estimates of COPD prevalence: Systematic review and meta-analysis. J Glob Health.

[17] Raghu G, Chen SY, Hou Q, Yeh WS (2016). & Collard, H. R. Incidence and prevalence of idiopathic pulmonary fibrosis in US adults 18-64 years old. Eur Respir J.

[18] Patterson KC, Strek ME (2013). Pulmonary fibrosis in sarcoidosis. Clinical features and outcomes. Ann Am Thorac Soc.

[19] Sartwell PE, Edwards LB (1974). Epidemiology of sarcoidosis in the U.S. Navy. Am J Epidemiol.

[20] Walker S (1998). Medium term results of lung transplantation for end stage pulmonary sarcoidosis. Thorax.

[21] Gutierrez C (2007). The effect of recipient’s age on lung transplant outcome. Am J Transplant.

[22] Sommer W (2015). Survival and spirometry outcomes after lung transplantation from donors aged 70 years and older. J Heart Lung Transplant.

[23] Callender CO (2009). Effect of donor ethnicity on kidney survival in different recipient pairs: an analysis of the OPTN/UNOS database. Transplant Proc.

[24] Egan TM (2006). Development of the new lung allocation system in the United States. Am J Transplant.

[25] Banga A, Sahoo D, Lane CR, Farver CF, Budev MM (2015). Disease Recurrence and Acute Cellular Rejection Episodes During the First Year After Lung Transplantation Among Patients With Sarcoidosis. Transplantation.

[26] Schultz HH (2014). Recurrence of sarcoid granulomas in lung transplant recipients is common and does not affect overall survival. Sarcoidosis Vasc Diffuse Lung Dis.

